# ToxCast on Target: *In Vitro* Assays and Computer Modeling Show Promise for Screening Chemicals

**DOI:** 10.1289/ehp.118-a172a

**Published:** 2010-04

**Authors:** Rebecca Clay Haynes

**Affiliations:** **Rebecca Clay Haynes** has written for *EHP* since 1993. Her work has also appeared on National Public Radio and in the *Christian Science Monitor* and *The Environmental Forum*. In addition, she is the author of two children’s science books related to astronomy and space exploration

Government agencies rely on toxicity data to help make key regulatory decisions on pesticides and other chemicals in the environment. But thousands of chemicals, including many currently in commerce, have yet to be tested for potential toxicity and impact on human diseases. Such testing can be time-consuming and expensive; a full set of regulatory tests for a single chemical may use thousands of animals and cost millions of dollars. To help address this large backlog of untested chemicals, the U.S. Environmental Protection Agency (EPA) recently completed the first phase of its large-scale ToxCast™ program, which looks at the potential for combining faster and less expensive *in vitro* assays with computer modeling to screen and prioritize chemicals for human toxicity **[*EHP*** **118:485–493; Judson et al.]**. This approach could both reduce the need for animal testing and speed up the regulatory process.

The ToxCast program evaluates the use of *in vitro* assays for understanding the types of molecular and pathway perturbations caused by chemical exposures and computer-based prioritization models for *in vivo* toxicity. In phase I, researchers chose 309 chemicals for which toxicity data were already available. Using 467 *in vitro* assays across 9 technologies, including high-throughput cell-free assays and cell-based assays in multiple human primary cells and cell lines, they investigated a broad spectrum of chemical activities at the molecular and pathway levels. Matching these *in vitro* results with existing data helped researchers build initial prioritization models for predicting toxicity of similar but untested chemicals.

This process also provided information on underlying mechanisms of toxicity, which are difficult to investigate directly using animal models. Using human cells or human cell constituents allowed researchers to measure the effects of chemicals on toxicity pathways that may be relevant to human disease. Based on the phase I examples, the ToxCast researchers feel confident that *in vitro* high-throughput data can help predict mechanisms of action for many other well-studied chemicals and indicate which other biological pathways may also be activated. This will lay the groundwork for screening untested chemicals and provide vital guidance for future testing.

The authors hope molecular and computational models will help better guide targeted testing of environmental contaminants but caution that building this new paradigm will itself take time and require input from multiple government organizations. They are launching a second phase of ToxCast to expand on and further confirm that *in vitro* testing can help predict human toxicity. Phase II could be completed over the next several years.

